# Incidence of Pancreas and Colorectal Adenocarcinoma in the US

**DOI:** 10.1001/jamanetworkopen.2025.4682

**Published:** 2025-04-14

**Authors:** Arvind Bussetty, Jing Shen, Petros C. Benias, Michael Ma, Molly Stewart, Arvind J. Trindade

**Affiliations:** 1 Department of Medicine, Rutgers University School of Medicine, New Brunswick, New Jersey; 2 Division of Gastroenterology, Rutgers University School of Medicine, New Brunswick, New Jersey; 3 RWJBarnabas Health System, Robert Wood Johnson University Hospital, New Brunswick, New Jersey

## Abstract

**Importance:**

Pancreas cancer is the seventh leading cause of cancer death and colorectal cancer is the second leading cause of cancer death worldwide, with increasing incidence for both, particularly in younger age cohorts.

**Objective:**

To report updated incidence trends for pancreas and colorectal adenocarcinomas in the US, with the specific intent of examining the annual percentage changes (APCs) in younger age groups.

**Design, Setting, and Participants:**

This retrospective cohort study used data from the Surveillance, Epidemiology, and End Results database to examine incidence trends from 2000 to 2021. Individuals with pancreatic and colorectal adenocarcinoma were included. Data through 2021 were released on April 17, 2024. Data analysis occurred from December 4, 2024, to February 1, 2025.

**Main Outcomes and Measures:**

Yearly incidence rates were obtained for pancreatic and colorectal adenocarcinoma. Temporal trends of pancreas adenocarcinoma incidence rates by covariates were measured by APC and 95% CIs.

**Results:**

From 2000 to 2021, 275 273 cases of pancreas adenocarcinoma were identified (142 633 male patients [51.8%]; 239 840 patients [87.1%] aged ≥55 years). The APC for pancreatic adenocarcinoma was 4.35 (95% CI, 2.03 to 6.73) in the group aged 15 to 34 years, which was statistically significantly higher than the APCs of 1.74 (95% CI, 1.59 to 1.89) (*P* = .007) for the group aged 55 years and older and 1.54 (95% CI, 1.18 to 1.90) (*P* = .004) for the group aged 35 to 54 years. A total of 1 215 200 cases of colorectal cancer were identified (641 776 male patients [52.8%]; 976 716 patients [80.4%] aged ≥55 years). The APC for colorectal adenocarcinoma was −3.31 (95% CI, −3.54 to −3.08) for the group aged 55 years and older, which was statistically significantly lower than the APCs of 1.75 (95% CI, 1.08 to 2.42) (*P* = .001) for the group aged 15 to 34 years and 0.78 (95% CI, 0.51 to 1.06) (*P* = .002) for the group aged 35 to 54 years.

**Conclusions and Relevance:**

The findings of this cohort study suggest that the incidence of pancreatic adenocarcinoma has increased among all age groups, whereas that of colorectal adenocarcinoma has increased among younger age groups. Clinicians should be aware of this trend when evaluating younger patients with relevant symptoms.

## Key Points

**Question:**

Did the incidence of pancreas and colorectal adenocarcinoma in the US increase between 2000 and 2021?

**Findings:**

In this cohort study of 275 273 cases of pancreas cancer and 1 215 200 cases of colorectal cancer, the highest annual percentage change for both adenocarcinomas occurred in the youngest age groups (ages 15-34 and 35-54 years).

**Meaning:**

These findings suggest that heightened awareness of this trend of increasing pancreas and colorectal cancer incidence is necessary when evaluating younger patients with possible corresponding symptoms.

## Teaser

This cohort study reports updated incidence trends for pancreas and colorectal adenocarcinomas, particularly annual percentage changes in younger age groups.

## Introduction

Pancreas cancer is the seventh leading cause of cancer death worldwide, has a 5-year survival rate of approximately 10%, and has doubled in incidence in the past 25 years.^1^ It was traditionally a cancer in elderly individuals, but data limited to 2018 have shown an increasing incidence in a younger population for the first time.^2,3^ Given that these reports were limited to 2018, it was unclear whether they represented a constant trend or were isolated onetime reports. Colorectal cancer is the second leading cause of cancer deathworldwide.^4^ Its incidence has been increasing in younger individuals per reports in 2021, leading to the lowering of the screening age from 50 to 45 years in average-risk, asymptomatic individuals.^5^ The aim of this study was to use a national cohort to determine updated incidence trends for pancreas and colorectal adenocarcinomas, with the specific intent of examining the annual percentage changes (APCs) in the younger age groups.

## Methods

For this cohort study, pancreatic and colorectal adenocarcinoma incidence rates per 100 000 population were obtained for the years 2000 to 2021 from the Surveillance, Epidemiology, and End Results (SEER) database, which includes limited-field data of 22 registries. The SEER database is a program of the National Cancer Institute that collects and publishes cancer incidence, prevalence, and mortality data from population-based cancer registries that cover approximately 47.9% of the US population. Data through 2021 were released on April 17, 2024.

Other forms of pancreas cancer were excluded (eg, squamous cell, mucinous cystadenocarcinoma, solid pseudopapillary carcinoma, serous cystadeocarcinoma, pancreatoblastoma, and neuroendocrine tumors) because these are rare and have varying biologic behavior. The Rutgers University institutional review board exempted the study, and informed consent was not needed owing to the deidentified nature of the data, in accordance with 45 CFR §46. This study followed the Strengthening the Reporting of Observational Studies in Epidemiology (STROBE) reporting guidelines for cohort studies.

### Statistical Analysis

Data analysis occurred from December 4, 2024, to February 1, 2025. The descriptive statistical analyses were used to present age-adjusted pancreas and colorectal adenocarcinoma incidence rates per 100 000 population by the covariates of age, sex, and race (American Indian or Alaska Native, Asian or Pacific Islander, Black, and White). Race and sex identification and reporting were provided by the SEER database. Data on race are included in this study to determine whether there are any significant race-associated incidence trends of which physicians should be aware. Temporal trends of pancreas and colorectal adenocarcinoma incidence rates by covariates were measured by APCs and 95% CIs using the Joinpoint Regression^6^ program version 5.3.0 (Statistical Research and Applications Branch, National Cancer Institute). Differences in mean APC across covariate groups were first assessed by 1-way analysis of variance, and further pairwise comparisons of groups showing statistical significance were performed by *t* tests^7^ using R statistical software version 4.4.1 (R Project for Statistical Computing). A 2-sided *P* < .05 was considered statistically significant.

## Results

**Figure 1.**
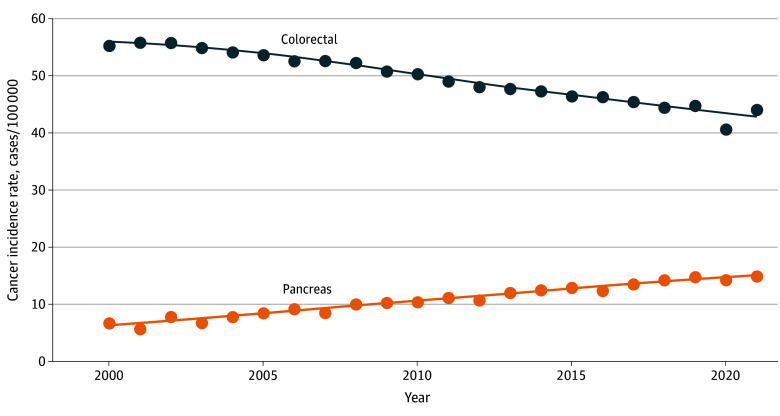
Overall Time Trend of Pancreas and Colorectal Cancer Incidence Rates

From 2000 to 2021, 275 273 cases of pancreas adenocarcinoma were identified (142 633 male patients [51.8%]; 239 840 patients [87.1%] aged ≥55 years) (Table). Joinpoint regression was used to analyze the incidence trends of pancreatic adenocarcinoma and colorectal adenocarcinoma from 2000 to 2021 in parallel, but no segmented changes were observed (Figure 1). There was an overall increasing incidence trend (Figure 1). Patient race was as follows: 14 920 Asian or Pacific Islander individuals (5.4%), 33 941 Black individuals (12.3%), 1161 American Indian or Alaska Native individuals (0.4%), and 225 251 White individuals (91.8%).

**Table.** Pancreas and Colorectal Adenocarcinoma Incidence Rates and Time Trend From 2000 to 2021, by Age, Sex, and Race

Covariate	Cancer cases, No. (%)	APC (95% CI)	*P* value	Comparison group
Pancreas cancer (n = 275 273)				
Age group, y^a^				
15-34	1633 (0.59)	4.35 (2.03 to 6.73)	004	35-54 y
35-54	33 800 (12.28)	1.54 (1.18 to 1.90)	.98	≥55 y
≥55	239 840 (87.13)	1.74 (1.59 to 1.89)	.007	15-34 y
Sex				
Male	142 633 (51.82)	1.87 (1.63 to 2.11)	.13	NA
Female	132 640 (48.18)	1.57 (1.27 to 1.87)		
Race				
American Indian or Alaska Native	1161 (0.42)	1.96 (1.09 to 2.83)	.32^b^	NA
Asian or Pacific Islander	14 920 (5.42)	1.71 (1.38 to 2.03)		
Black	33 941 (12.33)	1.52 (1.31 to 1.74)		
White	225 251 (81.83)	1.90 (1.80 to 2.00)		
Colorectal cancer (n = 1 215 200)				
Age group, y^c^				
15-34	13 893 (1.14)	1.75 (1.08 to 2.42)	.62	35-54 y
35-54	224 591 (18.48)	0.78 (0.51 to 1.06)	.002	≥55 y
≥55	976 716 (80.38)	−3.31 (−3.54 to −3.08)	.001	15-34 y
Sex				
Male	641 776 (52.81)	−2.70 (−2.91 to −2.48)	.52	NA
Female	573 424 (47.19)	−2.59 (−2.84 to −2.34)		
Race^d^				
American Indian or Alaska Native	6194 (0.51)	−1.56 (−2.04 to −1.07)	.001	Asian or Pacific Islander
			.001	Black
			.001	White
Asian or Pacific Islander	75872 (6.24)	−2.71 (−2.95 to −2.47)	.86	Black
			.77	White
Black	148 050 (12.18)	−2.87 (−3.16 to −2.58)	.99	NA
White	985 082 (81.07)	−2.96 (−3.17 to −2.75)		

Abbreviations: ANOVA, analysis of variance; APC, annual percentage change; NA, not applicable.

^a^ One-way ANOVA, *F* = 7.06, and *P* = .002.

^b^ One-way ANOVA, *F* = 1.19.

^c^ One-way ANOVA, *F* = 11.53, and *P* = .001.

^d^ One-way ANOVA, *F* = 19.78, and *P* = .001.

From the years of 2000 to 2021, 1 215 200 cases of colorectal adenocarcinoma were identified (641 776 male patients [52.8%]; 976 716 patients aged ≥55 years [80.4%]) (Table). There was an overall decreasing incidence trend (Figure 1). Patient race was as follows: 75 872 Asian or Pacific Islander individuals (6.2%), 148 050 Black individuals (12.2%), 6194 American Indian or Alaska Native individuals (0.5%), and 985 082 White individuals (81.1%).

**Figure 2.**
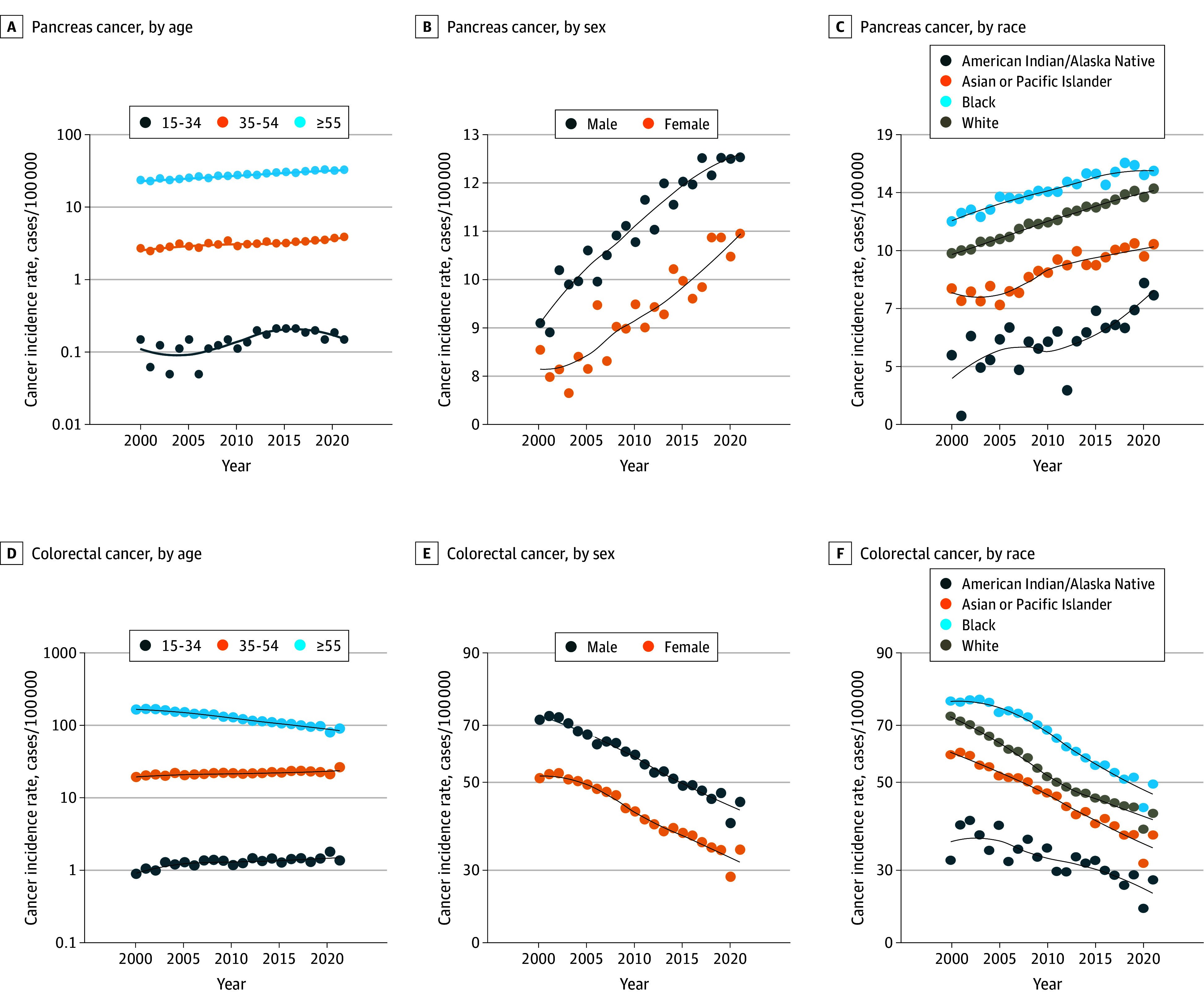
Time Trend of Pancreas and Colorectal Cancer Incidence Rates by Age, Sex, and Race Pancreas cancer incidence rates are shown by age (A), sex (B), and race (C). Colorectal cancer incidence rates are shown by age (D), sex (E), and race (F).

The APC for pancreatic adenocarcinoma was highest in the group aged 15 to 34 years (4.35; 95% CI, 2.03-6.73), which was statistically significantly higher than the APC for the group aged 55 years and older (1.74; 95% CI, 1.59-1.89) (*P* = .007) and that for the group aged 35 to 54 years (1.54; 95% CI, 1.18-1.90) (*P* = .004). The Table and Figure 2 show the age group trends. There was no statistically significant difference between the APCs for age groups 35 to 54 years and 55 years and older (*P* = .98). The APC for male patients (1.87; 95% CI, 1.63-2.11) was higher than that for female patients (1.57; 95% CI, 1.27-1.87) but the difference was not statistically significant (*P* = .13). The APCs were increased among all racial groups (Table and Figure 2).

The APC for colorectal adenocarcinoma was −3.31 (95% CI, −3.54 to −3.08) for the group aged 55 years and older, which was statistically significantly lower than the APCs of 1.75 (95% CI, 1.08 to 2.42) (*P* = .001) for the group aged 15 to 34 years and 0.78 (95% CI, 0.51 to 1.06) (*P* = .002) for the group aged 35 to 54 years (Table). The downward trending APC for male patients (−2.70; 95% CI, −2.91 to −2.48) was greater than that for female patients (−2.59; 95% CI, −2.84 to −2.34) but the difference was not statistically significant (*P* = .52). The APCs were decreasing among all racial groups (Table and Figure 2). Further subgroup analysis regarding age group, sex, and race is provided in the eTable and eFigures 1 and 2 in Supplement 1.

## Discussion

In this cohort study, we analyzed data from the SEER database examining the incidence of pancreatic and colorectal adenocarcinoma from 2000 to 2021. In contrast to colorectal adenocarcinoma, which overall had a decreasing incidence, pancreatic adenocarcinoma appeared to be increasing among all age groups. Although the highest incidence of pancreatic adenocarcinoma was still in the older age group of 55 years and older, there was an alarming increase of pancreatic adenocarcinoma in the youngest age group of 15 to 34 years of age, with an APC of 4.35 (95% CI, 2.03-6.73). It is unclear what factors are underlying the increased incidence in this age group, but they may include such social behaviors as smoking and alcohol consumption.^3^

In contrast to pancreatic adenocarcinoma, the incidence of colorectal adenocarcinoma was overall decreasing. This was primarily associated with the decreasing incidence in the oldest group aged 55 years and older (APC −3.31; 95% CI, −3.54 to −3.08). However, the incidence in the youngest group aged 15 to 34 years was also increasing, with an APC of 1.75 (95% CI, 1.08 to 2.42). In the period of this study, most colorectal cancer screening started at age 50 years. It is likely that increased screening efforts through colonoscopy, stool DNA, or fecal immunohistochemistry testing are driving these rates down. With the recent lowering of age qualification for colorectal cancer screening to age 45 years, it is likely we will see a decline in colorectal adenocarcinoma incidence for the group aged 35 to 54 years.

This is not the first study to show the increasing incidence of colorectal adenocarcinoma in the younger age groups.^5,8^ However, per our literature review, this does appear to be the first SEER analysis showing this, and is the most up to date with data up to 2021. This is also not the first study for pancreatic cancer showing these findings. Two previous studies using the SEER database showed similar findings.^2,9^ Key differences are that we limited our study to adenocarcinoma (given this is the most aggressive and common form of pancreatic cancer), we provided incidence data by race, we provided a comparison with colorectal cancer incidence from the same database, and most importantly we provided an updated landscape of incidence data with 3 additional years showing the worrisome increasing incidence of pancreatic adenocarcinoma among all age groups.

### Limitations

One of the limitations of the study is that 47.9% of the population is represented in the database. However, the SEER database is designed to allow for accurate characterization of cancer incidence trends in the entire population. Coding of SEER has been reported as reliable for common cancers such as pancreatic and colorectal adenocarcinoma but less so for uncommon cancers.^10^ By limiting this report to adenocarcinoma, which is the most common type of pancreas cancer, we are more likely to have a homogeneous and accurate study group. Including rare pancreas tumors would create a heterogeneous study group.

## Conclusions

In conclusion, we show in this national cohort the worrisome trend of increasing pancreatic adenocarcinoma in all age groups and the increasing incidence of colorectal adenocarcinoma in the youngest age groups. Heightened awareness of this trend is necessary when evaluating younger patients with possible corresponding symptoms.

## Notes

Supplement 1. **eTable.** Pancreas and colorectal cancer incidence rates and time-trend from 2000 to 2021 years by two co-variates **eFigure 1.** Pancreas and colorectal cancer incidence rates by age and gender **eFigure 2.** Pancreas and colorectal cancer incidence rates by gender and race and ethnicity

Supplement 2.
Data Sharing Statement

## References

[1] Ramai D, Smith ER, Wang Y, . Epidemiology and socioeconomic impact of pancreatic cancer: an analysis of the Global Burden of Disease Study 1990-2019. Dig Dis Sci. 2024;69(4):1135-1142. doi:10.1007/s10620-024-08292-1 38383939

[2] Gaddam S, Abboud Y, Oh J, . Incidence of pancreatic cancer by age and sex in the US, 2000-2018. JAMA. 2021;326(20):2075-2077. doi:10.1001/jama.2021.18859 34689206 PMC8543346

[3] Abboud Y, Samaan JS, Oh J, . Increasing pancreatic cancer incidence in young women in the United States: a population-based time-trend analysis, 2001-2018. Gastroenterology. 2023;164(6):978-989.e6. doi:10.1053/j.gastro.2023.01.022 36775072 PMC11364483

[4] Shaukat A, Kahi CJ, Burke CA, Rabeneck L, Sauer BG, Rex DK. ACG clinical guidelines: colorectal cancer screening 2021. Am J Gastroenterol. 2021;116(3):458-479. doi:10.14309/ajg.0000000000001122 33657038

[5] Davidson KW, Barry MJ, Mangione CM, ; US Preventive Services Task Force. Screening for colorectal cancer: US Preventive Services Task Force Recommendation Statement. JAMA. 2021;325(19):1965-1977. doi:10.1001/jama.2021.6238 34003218

[6] Kim HJ, Chen HS, Byrne J, Wheeler B, Feuer EJ. Twenty years since Joinpoint 1.0: two major enhancements, their justification, and impact. Stat Med. 2022;41(16):3102-3130. doi:10.1002/sim.9407 35522060

[7] Clegg LX, Hankey BF, Tiwari R, Feuer EJ, Edwards BK. Estimating average annual per cent change in trend analysis. Stat Med. 2009;28(29):3670-3682. doi:10.1002/sim.3733 19856324 PMC2843083

[8] Siegel RL, Fedewa SA, Anderson WF, . Colorectal cancer incidence patterns in the United States, 1974-2013. J Natl Cancer Inst. 2017;109(8):djw322. doi:10.1093/jnci/djw322 28376186 PMC6059239

[9] Gordon-Dseagu VL, Devesa SS, Goggins M, Stolzenberg-Solomon R. Pancreatic cancer incidence trends: evidence from the Surveillance, Epidemiology and End Results (SEER) population-based data. Int J Epidemiol. 2018;47(2):427-439. doi:10.1093/ije/dyx232 29149259 PMC5913617

[10] Park HS, Lloyd S, Decker RH, Wilson LD, Yu JB. Limitations and biases of the Surveillance, Epidemiology, and End Results database. Curr Probl Cancer. 2012;36(4):216-224. doi:10.1016/j.currproblcancer.2012.03.011 22481009

