# Supplementary material for: Incidence of Pancreas and Colorectal Adenocarcinoma in the US
Source: JAMA Netw Open. 2025 Apr 14;8(4):e254682. doi: 10.1001/jamanetworkopen.2025.4682 (PMC11997727; doi:10.1001/jamanetworkopen.2025.4682)
Supplement: Supplement 1. — eTable. Pancreas and colorectal cancer incidence rates and time-trend from 2000 to 2021 years by two co-variates eFigure 1. Pancreas and colorectal cancer incidence rates by age and gender eFigure 2. Pancreas and colorectal cancer incidence rates by gender and race and ethnicity [file jamanetwopen-e254682-s001.pdf]

Supplemental Online Content

Bussetty A, Shen J, Benias PC, Ma M, Stewart M, Trindade AJ. Incidence of pancreas and colorectal adenocarcinoma in the US. *JAMA Netw Open*. 2025;8(4):e254682. doi:10.1001/jamanetworkopen.2025.4682

eTable. Pancreas and colorectal cancer incidence rates and time-trend from 2000 to 2021 years by two co-variates
eFigure 1. Pancreas and colorectal cancer incidence rates by age and gender
eFigure 2. Pancreas and colorectal cancer incidence rates by gender and race and ethnicity

This supplemental material has been provided by the authors to give readers additional information about their work.

© 2025 Bussetty A et al. *JAMA Network Open*.

eTable. Pancreas and colorectal cancer incidence rates and time-trend from 2000 to 2021 years by two co-variates

Co-variate	Gender	Pancreas cancer cases (N=275273)	APC (95% CI) *	P value
**Age group (yrs)**				
15-34	Male	797 (0.29%)	5.46 (2.20-8.82)	
	Female	836 (0.30%)	3.47 (0.89-6.13)	0.36
35-54	Male	19428 (7.06%)	1.31 (0.74-1.88)	
	Female	14372 (5.22%)	1.89 (1.41-2.37)	0.13
55 and up	Male	122408 (44.47%)	1.92 (1.67-2.18)	
	Female	117432 (42.66%)	1.53 (1.20-1.86)	0.07
**Race**				
American Indian/Alaska Native	Male	602 (0.22%)	3.60 (1.84-5.39)	
	Female	559 (0.20%)	0.44 (-1.25 to 2.17)	0.02
Asian or Pacific Islander	Male	7327 (2.66%)	1.64 (1.25-2.03)	
	Female	7593 (2.76%)	1.79 (1.40-2.18)	0.60
Black	Male	15973 (5.80%)	1.43 (1.17-1.68)	
	Female	17968 (6.53%)	1.63 (1.39-1.88)	0.27
White	Male	118731 (43.13%)	1.84 (1.73-1.96)	
	Female	106520 (38.70%)	1.97 (1.87-2.07)	0.10
**Co-variate**	**Gender**	**Colon & Rectum cancer cases (N=1215200)**	**APC (95% CI) ***	**P value**
**Age group (yrs)**				
15-34	Male	7056 (0.58%)	1.83 (0.87-2.81)	
	Female	6837 (0.56%)	1.81 (0.85-2.78)	0.98
35-54	Male	121569 (10.00%)	0.92 (0.65-1.18)	
	Female	103022 (8.48%)	0.63 (0.27-0.99)	0.21
55 and up	Male	513151 (42.23%)	-3.33 (-3.56 to -3.09)	
	Female	463565 (38.15%)	-3.29 (-3.55 to -3.02)	0.83
**Race**				
American Indian/Alaska Native	Male	3191 (0.26%)	-1.60 (-2.20 to -0.99)	
	Female	3005 (0.25%)	-1.51 (-2.08 to -0.93)	0.83
Asian or Pacific Islander	Male	40336 (3.32%)	-2.73 (-2.98 to -2.49)	
	Female	35536 (2.92%)	-2.68 (-2.98 to -2.37)	0.80

© 2025 Bussetty A et al. *JAMA Network Open*.

Black	Male	73594 (6.06%)	-2.82 (-3.11 to -2.62)	
	Female	74456 (6.13%)	-2.95 (-3.28 to -2.62)	0.54
White	Male	524655 (43.17%)	-3.10 (-3.33 to -2.87)	
	Female	460427 (37.89%)	-2.77 (-2.96 to -2.58)	0.04

* APC: annual percentage change

© 2025 Bussetty A et al. *JAMA Network Open*.

eFigure 1. Pancreas and colorectal cancer incidence rates by age and gender

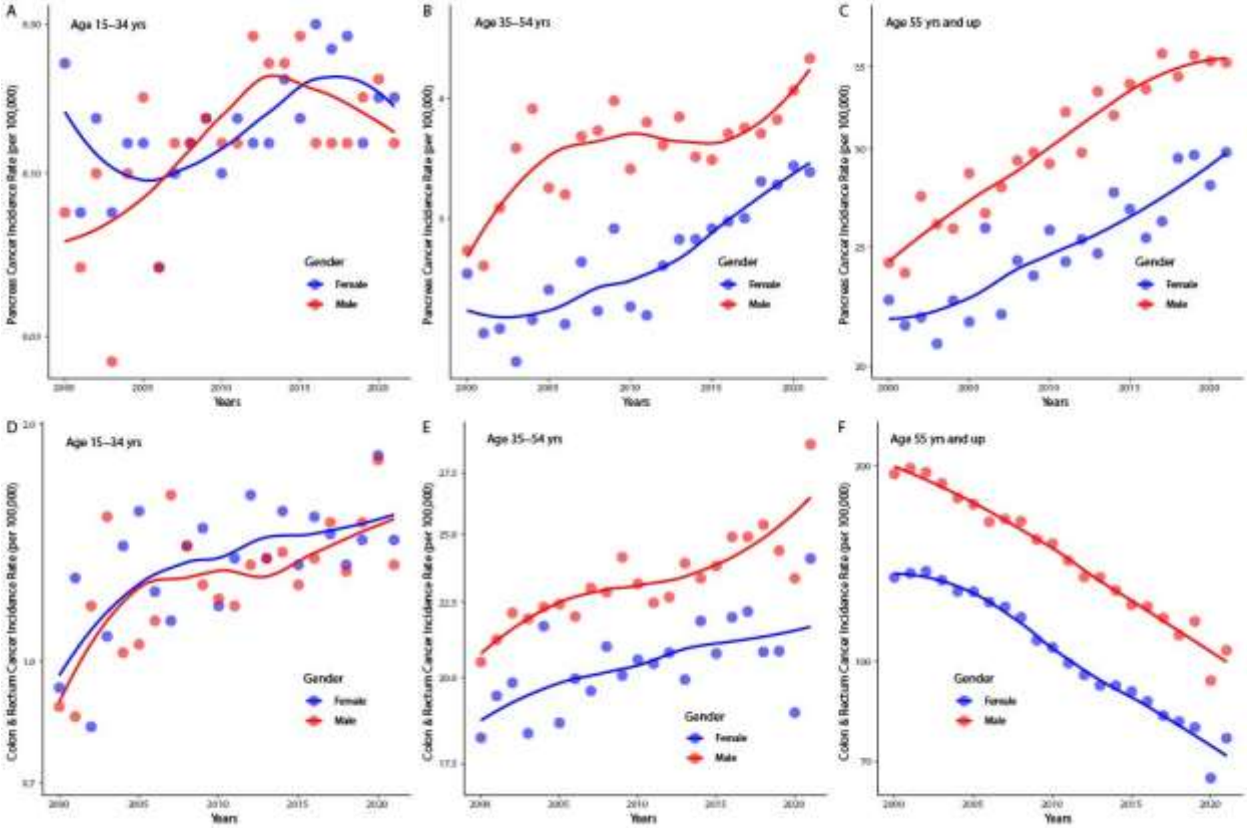

© 2025 Bussetty A et al. *JAMA Network Open*.

eFigure 2. Pancreas and colorectal cancer incidence rates by gender and race and ethnicity

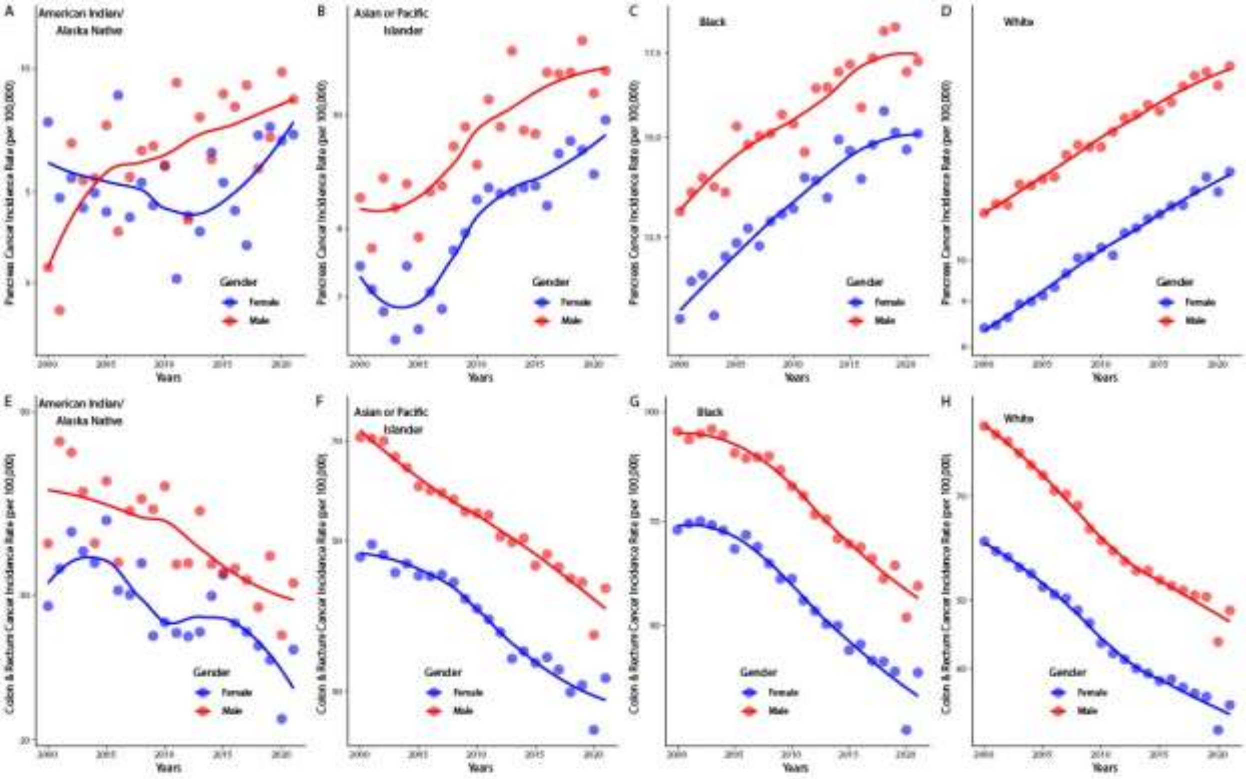

© 2025 Bussetty A et al. *JAMA Network Open*.
