# Supplementary material for: Incidence of Pancreas and Colorectal Adenocarcinoma in the US
Source: JAMA Netw Open. 2025 Apr 14;8(4):e254682. doi: 10.1001/jamanetworkopen.2025.4682 (PMC11997727; doi:10.1001/jamanetworkopen.2025.4682)
Supplement: Supplement 2. — Data Sharing Statement [file jamanetwopen-e254682-s002.pdf]

Data Sharing Statement

Bussetty. Incidence of Pancreas and Colorectal Adenocarcinoma in the US. *JAMA Netw Open*. Published April 14, 2025. doi:10.1001/jamanetworkopen.2025.4682

Data
Data available: Yes
Data types: Deidentified participant data
How to access data: arvind.trindade@gmail.com
When available: With publication

Supporting Documents
Document types: None

Additional Information
Who can access the data: researchers whose proposed use of the data has been approved
Types of analyses: for a specified purpose
Mechanisms of data availability: with a signed data access agreement
